# Supplementary material for: A capsular myofibroblastic niche maintains hematopoietic stem cells in the spleen
Source: EMBO J. 2025 Jun 5;44(14):3983–4012. doi: 10.1038/s44318-025-00477-2 (PMC12264044; doi:10.1038/s44318-025-00477-2)
Supplement: Supplementary file 1 — Appendix [file 44318_2025_477_MOESM1_ESM.pdf]

Appendix

A capsular myofibroblastic niche maintains hematopoietic stem cells in the spleen

Mehatre SH *et al.*

Corresponding author: Satish Khurana, satishkhurana@iisertvm.ac.in

Serial no.	Table of contents	Page number
1	Appendix Figure S1	1
2	Appendix Figure S2	2
3	Appendix Figure S3	3
4	Appendix Table S1	4
5	Appendix Table S1	5

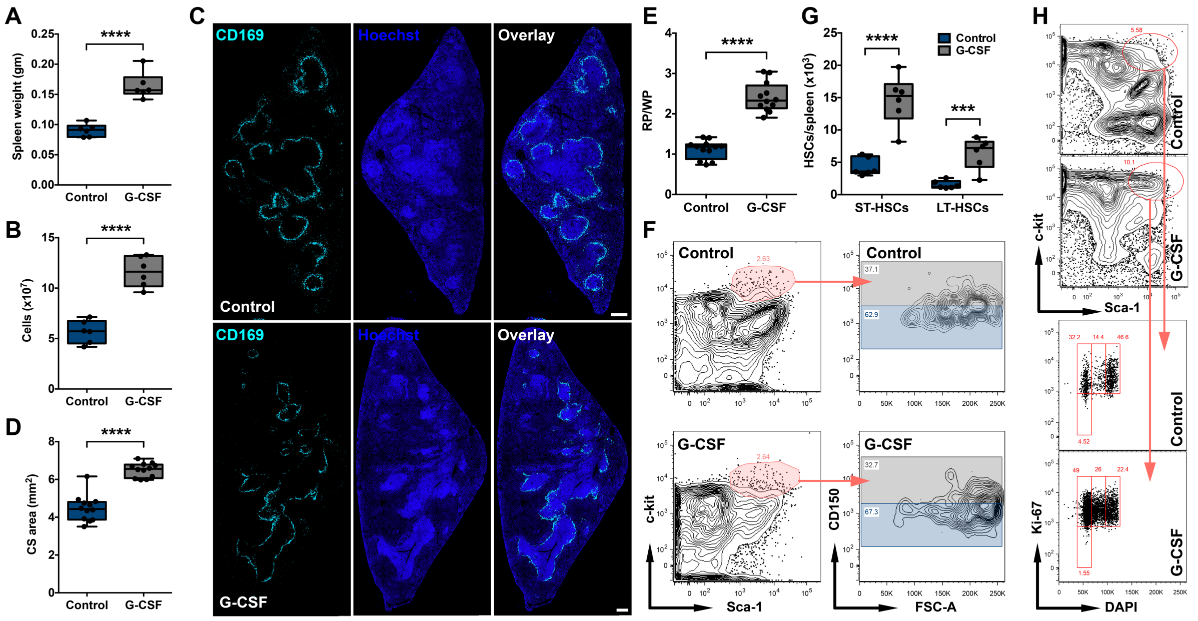

Appendix Figure S1. G-CSF treatment leads to morphological changes in the splenic architecture

(**A**) Effect of G-CSF treatment on spleen weight. Spleen tissues were harvested after 5 daily doses of G-CSF were given and weight of the entire spleen was compared with controls (n=5). (**B**) Comparison of total spleen cellularity with and without G-CSF treatment. MNCs were harvested from spleen tissues without enzymatic treatment, and viable cell counts were taken after RBC lysis using a Neubauer chamber (n=6). (**C**) Immunostaining to detect RP and WP areas in spleen tissues with and without G-CSF treatment. The analysis was based on immunolocalization of CD169$^+$ marginal zone macrophages (scale bar=200 µm; n=4, N=12 tile scans). (**D**) Change in the total CS area of spleen tissue following G-CSF treatment. The area was measured from the spleen sections immunostained for CD169 and counterstained for nuclei with Hoechst 33342 (n=4, N=12 tile scans). (**E**) Comparison of the ratio of RP and WP area in spleen tissues obtained from mice treated with or without G-CSF. WP areas were detected by the presence of marginal zone macrophages identified by immunostaining for CD169. RP areas were quantified exclusive of WP, vasculature, trabecular, and capsular areas (n=4, N=12 tile scans). (**F**) Flowcytometry analysis to examine LT-HSC (Lin$^-$CD41$^-$CD48$^-$Sca-1$^+$c-kit$^+$CD150$^+$ cells) and ST-HSC (Lin$^-$CD41$^-$CD48$^-$Sca-1$^+$c-kit$^+$CD150$^-$ cells) populations in splenic MNCs harvested from control (same data used for Fig. 7G) and G-CSF treated mice. (**G**) Comparison of total LT-HSC and ST-HSC in spleen tissues treated with and without G-CSF (n=6). (**H**) Flow cytometry analysis is used to compare the cell cycle status of HSCs (Lin$^-$CD41$^-$CD48$^-$Sca-1$^+$c-kit$^+$ cells) in the spleen with and without G-CSF treatment (same samples were used for data presented in Fig. 7E and Fig. EV3X). DAPI and Ki-67 based analysis for G_0, G_1, S and G_2M stages of cell cycle was performed along with immunostaining for HSC markers. Data is presented as box-whiskers plot (median with min to max) in panels (A,B,D,E,G). The p-value in figures (A,B,D,E,G) were calculated by the Student's *t*-test, *** p<0.001, **** p<0.0001.

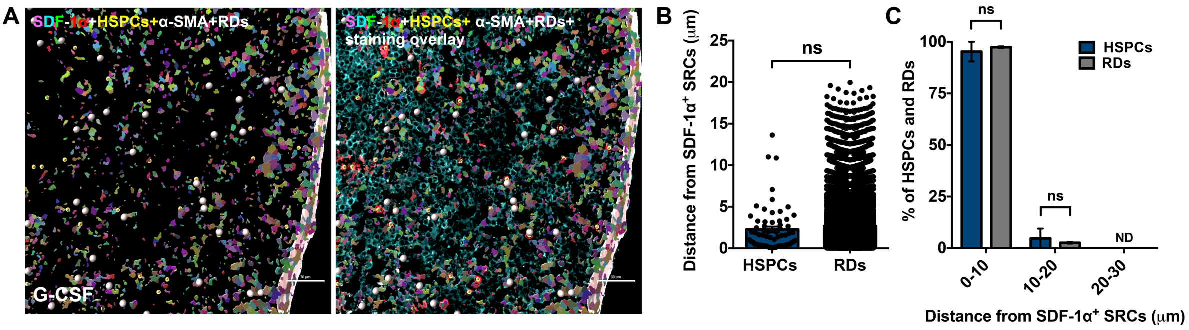

Appendix Figure S2. HSPCs associate specifically with capsular myofibroblasts

(**A**) Confocal based imaging for spleen tissue sections immunostained for the localization of HSPCs (identified as c-kit^{+}lin^{-}CD48^{-}CD41^{-} cells) along with SDF-1α and α-SMA. Pseudo-surfaces for HSPCs and α-SMA^{+} myofibroblasts, random dots, and heatmaps for SDF-1α expression were generated on Imaris. Euclidean distances for HSPCs (illuminated yellow) and RDs with respect to the nearest observable SDF-1α^{+} splenic red pulp cell (SRC; based on the pseudo-surfaces generated) were determined. (**B**) Comparison of Euclidean distances measured for HSPCs and RDs with reference to the nearest observable of α-SMA^{+} SRCs in splenic sections after G-CSF treatment (n=4, N=64 HSPCs; each dot represents an HSPC or an RD). (**C**) Distribution frequency of HSPCs and RDs in splenic tissue at sequential intervals of 10 µm, relative to the SDF-1α^{+} SRCs (n=4, N=64 HSPCs). Data is presented as bar graph (mean±SEM) in panels (B,C). The p-value in figures (B,C) were calculated by the Student's *t*-test, ns p>0.05, and ND indicates not detected.

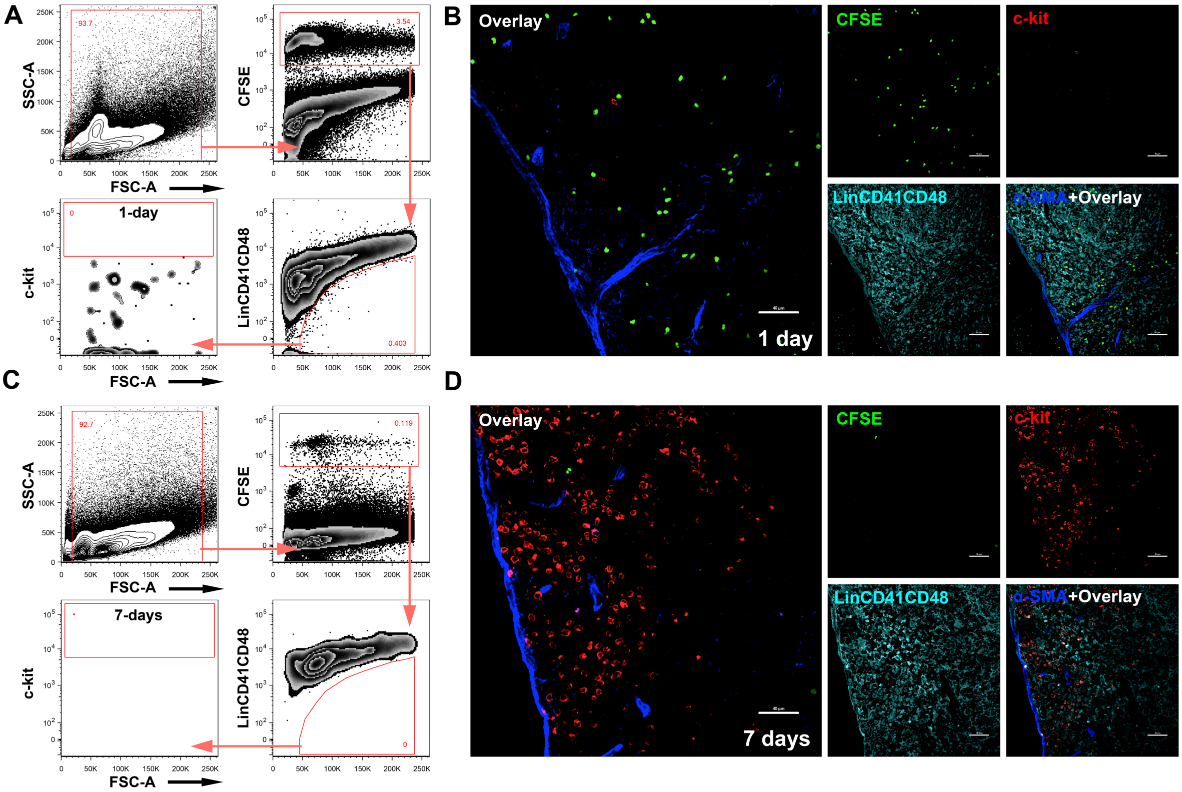

Appendix Figure S3. Transplanted HSPCs do not home in the splenic hematopoietic niche upto 8 days of 5-FU treatment

(A) Flow cytometry analysis to detect transplanted CFSE$^+$ HSPC (Lin$^-$CD41$^-$CD48$^-$c-kit$^+$) populations within the MNCs from a spleen of 5-FU treated mice one day after transplantation with CFSE labelled lineage-depleted BM cells. **(B)** Confocal images of spleen CS immunostained to localize transplanted CFSE$^+$ HSPCs one day after transplantation (scale bar=40 µm). **(C)** Flow cytometry analysis to examine transplanted CFSE$^+$ HSPC population within the MNCs from the spleen tissue of 5-FU treated mice seven days after transplantation. **(D)** Confocal images of spleen CS immunostained to localize CFSE$^+$ transplanted HSPCs 7 days after transplantation (scale bar=50 µm).

Appendix Table S1. List of primers used in this study

Sr. No.	Primer name	Sequence 5'-3'
1	Mm-*Gapdh*-F	ACCCAGAAGACTGTGGATGG
	Mm-*Gapdh*-R	TTCAGCTCTGGGATGACCTT
2	Mm-*Ang1*-F	TGCAAAGGGATGCTCCACACGT
	Mm-*Ang1*-R	AGCATGGTGGCCGTGTGGTTT
3	Mm-*Vcam-1*-F	GCCACCCTCACCTTAATTGC
	Mm-*Vcam-1*-R	CAGCACACGTCAGAACAACC
4	Mm-*Epo*-F	CCACCCTGCTGCTTTTACTC
	Mm-*Epo*-R	CTCAGTCTGGGACCTTCTGC
5	Mm-*Ccl2*-F	CCTGCTGTTCACAGTTGCCG
	Mm-*Ccl2*-R	CACAGACCTCTCTCTTGAGCTT
6	Mm-*Scf*-F	AGCCAGAGTCCTTCAGAGAGAT
	Mm-*Scf*-R	CATAACGCACTAGGTTTGCCG
7	Mm-*Tpo*-F	GGAGAAAGCTTTAAACAAGAGGC
	Mm-*Tpo*-R	GGCCCTTCCCTAACACTGAC
8	Mm-*Il-6*-F	AGCCAGAGTCCTTCAGAGAGAT
	Mm-*Il-6*-R	CATAACGCACTAGGTTTGCCG
9	Mm-*Postn*-F	AGTTTGTTCGTGGCAGCACCTT
	Mm-*Postn*-R	TCGTCATTGCAGGTCCTTCCGT
10	Mm-*Icam-1*-F	ACCTGCACTTTGCCCTGGCC
	Mm-*Icam-1*-R	ACCCTGGGGCAGGAAGGCTT
11	Mm- *Sdf-1α*-F	GGAGAAAGCTTTAAACAAGAGGC
	Mm- *Sdf-1α*-R	GGCCCTTCCCTAACACTGAC

Appendix Table S2. List of antibodies used in this study

Sr. No.	Antibodies	Source	Catalog number
1	BB700 conjugated anti-mouse Sca-1 (Ly6A/E)	BD Pharmingen	742089
2	PECy7 conjugated anti-mouse CD150	Biolegend	115914
3	PE conjugated anti-mouse c-kit (CD117)	Biolegend	105808
4	FITC conjugated anti-mouse CD48 FITC	BioLegend	103404
5	APC conjugated anti-mouse Lineage Antibody Cocktail	BD Pharmingen	558074
6	APC conjugated anti-mouse CD11b	Biolegend	101212
7	APC conjugated anti-mouse Gr-1 (Ly6G)	Biolegend	108412
8	APC conjugated anti-mouse CD4	Biolegend	100516
9	APC conjugated anti-mouse CD8a	Biolegend	100712
10	FITC conjugated anti-mouse CD45R/B220	Biolegend	103206
11	AF647 conjugated anti-mouse Ki-67	BD Pharmingen	561126
12	FITC conjugated anti-mouse Ter119	BD Pharmingen	557915
13	FITC conjugated anti-mouse Gr-1/Ly-6G/C	BD Pharmingen	553126
14	FITC conjugated anti-mouse CD3e	Invitrogen	11-0031-85
15	FITC conjugated anti-mouse CD41	BD Pharmingen	561849
16	AF488 conjugated anti-mouse F4/80	BD Pharmingen	567201
17	FITC conjugated anti-mouse CD45	Biolegend	103108
18	AF647 conjugated anti-mouse B220	Biolegend	103226
19	Anti-mouse α-SMA	Abcam	ab5694
20	Anti-mouse α-SMA	R & D	MAB1420
21	Anti-mouse CD31	Abcam	Ab28364
22	AF488 conjugated anti-mouse Lyve-1	eBioscience	53-0443-82
23	Anti-mouse CD169	Biolegend	142419
24	Anti-mouse Ter119	R & D	MAB1125
25	Anti-mouse Gr-1	R & D	MAB1037
26	Anti-mouse CD3e	R & D	MAB4841
27	Anti-mouse CD41	R & D	MAB4118
28	Anti-mouse CD48	R & D	MAB3327
29	Anti-mouse F4/80	R & D	MAB5580
30	Anti-mouse CD11b	R & D	MAB1124

Sr. No.	Antibodies	Source	Catalog number
31	Anti-mouse c-kit	R and D	AF1356
32	Anti-mouse CD150	Abcam	ab156288
33	Anti-mouse CD150	Biolegend	115908
34	Anti-mouse SDF-1α	Abcam	ab9797
35	Anti-mouse Ki-67	eBioscience	13-5698-82
36	Anti-mouse CD45	eBioscience	13-0451-85
37	Fab Fragment	Jackson ImmunoResearch	115-007-003
