# Supplementary material for: A capsular myofibroblastic niche maintains hematopoietic stem cells in the spleen
Source: EMBO J. 2025 Jun 5;44(14):3983–4012. doi: 10.1038/s44318-025-00477-2 (PMC12264044; doi:10.1038/s44318-025-00477-2)
Supplement: Supplementary file 3 — Expanded View Figures [file 44318_2025_477_MOESM3_ESM.pdf]

Expanded View Figures

Figure EV1. Capsular myofibroblasts constitute to the hematopoietic niche in adult spleen.

(**A**) Flow cytometry was performed to evaluate different marker combinations for identifying primitive HSCs (pHSCs or $CD150^+CD41^-CD48^-$LSK cells) using three fluorophores. The $Lin^-CD41^-CD48^-$ (or 3^-) cells (**Ai**) were further gated on $CD150^+$c-kit^+ (**Aii**), $CD150^+$Sca-1^+ (**Aiii**), and Sca-1^+c-kit^+ (**Aiv**) cells; and the proportion of pHSCs in each one of them was examined ($n = 7$). (**B**) Representative confocal images showing immunofluorescence-based localization of 3^-c-kit^+ HSPCs, along with α-SMA^+ capsular myofibroblasts. Pseudo surfaces for HSPCs (illuminated yellow) and capsular myofibroblast (illuminated white) were generated using Imaris. The Euclidean distance from the surfaces of HSPCs with respect to the nearest observable capsular myofibroblast in the spleen was determined. Scale bars = 20 µm (left panel), 3 µm (right panel). (**C**) Spatial distribution frequency of HSPCs in sequential intervals of 100 µm relative to capsular myofibroblasts ($n = 4$; $N = 20$ images). (**D**) Confocal based immunofluorescence imaging to locate 3^-c-kit^+ HSPCs along with α-SMA^+ capsular myofibroblasts. Pseudo surfaces for HSPCs (illuminated yellow) and capsular myofibroblast (illuminated white) were generated using Imaris. An equivalent number of RDs, as that of HSPCs identified, were generated, and 100 iterations were performed for analysis. The Euclidean distances from the HSPC cell surfaces and RDs, with respect to the nearest observable capsular myofibroblast in the spleen were determined (scale bar = 20 µm). (**E**) Comparison of Euclidean distances measured for HSPCs and RDs with reference to the nearest observable of α-SMA^+ capsular myofibroblast in splenic sections ($n = 3$, $N = 818$ HSPCs; each dot represents an HSPC or an RD). (**F**) Distribution of HSPCs and RDs at sequential intervals (30 µm each) from the splenic capsule identified by α-SMA immunostaining. The HSPCs were identified as 3^-c-kit^+ cells in the splenic tissue, and the surfaces and RDs were generated using Imaris ($n = 3$, $N = 818$ HSPCs). (**G**) The proportion of Ki-67^- (quiescent) and Ki-67^+ (proliferative) cells with pHSC and HSPC populations identified by confocal based imaging of immunostained spleen sections ($n = 4$, N; pHSCs = 557, HSPCs = 670). Data is presented as bar graph (mean ± SEM) in panel (**F**) or box-whiskers plot (median with min to max) in panels (**C**, **E**) or stacked bars in (**G**). The p-value in figures (**C**–**F**) were calculated by the Student's t-test, $*p < 0.05$, $**p < 0.01$, $****p < 0.0001$, and ns $p > 0.05$.

© The Author(s)

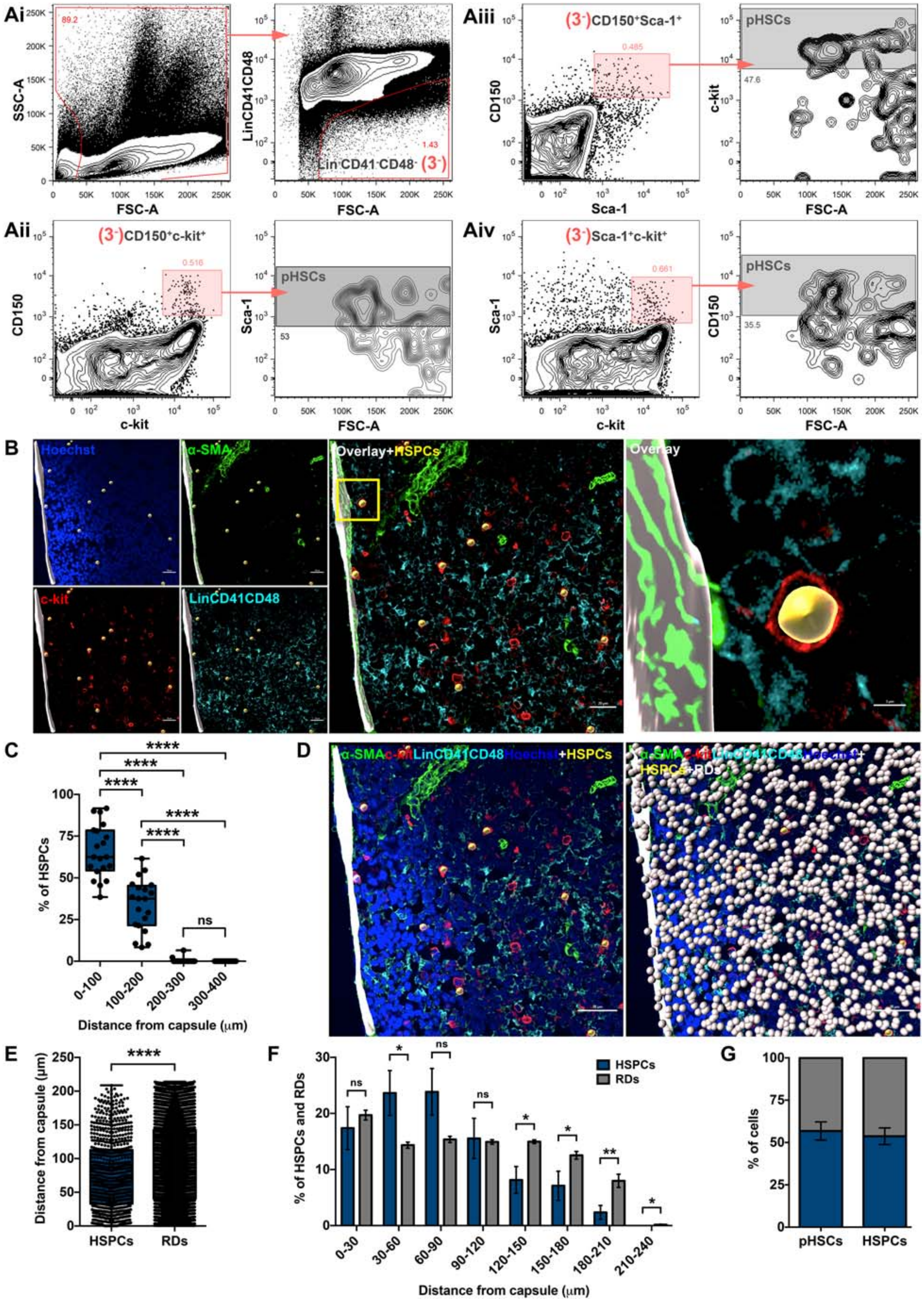
Ai
SSC-A
FSC-A
LinCD41CD48
Lin-CD41-CD48- (3-)
Aii
(3-)CD150+c-kit+
CD150
c-kit
Sca-1
pHSCs
Aiii
(3-)CD150+Sca-1+
Sca-1
pHSCs
Aiv
(3-)Sca-1+c-kit+
B
Hoechst
α-SMA
c-kit
LinCD41CD48
Overlay+HSPCs
Overlay
C
% of HSPCs
Distance from capsule (μm)
D
α-SMAc-kitLinCD41CD48Hoechst+HSPCs
E
Distance from capsule (μm)
HSPCs
RDs
F
% of HSPCs and RDs
HSPCs
RDs
Distance from capsule (μm)
G
% of cells
pHSCs
HSPCs

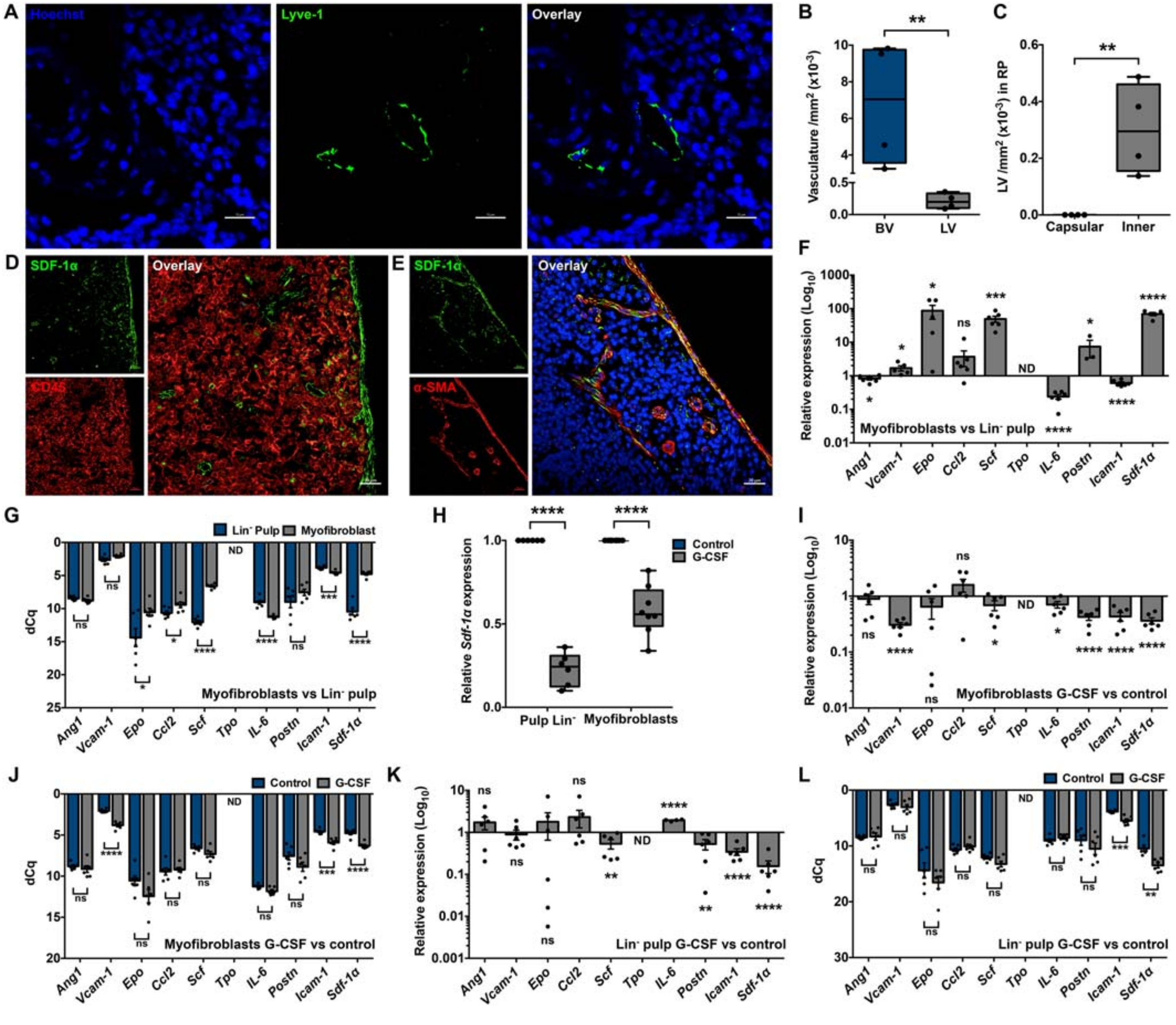

Figure EV2. G-CSF infusion leads to alterations of hematopoietic niche factors in spleen.

(**A**) Immunostaining was performed to detect lymphatic vessels in the spleen using antibodies against lymphatic vessel endothelial hyaluronan receptor 1 (Lyve-1). Counterstaining was performed with Hoechst 33342 and confocal based imaging was performed (scale bar = 15 µm). Splenic CS is divided into 200 µm wide peripheral capsular zone and inner core. (**B**) Comparison of blood and lymphatic vessel area in splenic tissue normalized to total splenic CS area ($n = 4$). (**C**) Comparison of vascular distribution of lymphatic vessels in the 200 µm wide peripheral capsular zone and inner core area in the RP of splenic CS ($n = 4$). (**D**) Confocal microscopy was performed to locate SDF-1α expression in the spleen tissue with reference to hematopoietic and non-hematopoietic fractions identified based on the expression pan-leukocyte antigen CD45 (scale bar = 20 µm). (**E**) Immunodetection of SDF-1α expression in splenic capsular and trabecular myofibroblasts identified by α-SMA expression. Confocal images show small vessels and trabecular area in the splenic section with immunostaining performed for SDF-1α along with α-SMA and nuclear staining with Hoechst 33342 (scale bar = 20 µm). (**F**) Detection and comparison of transcript levels of known hematopoietic regulators *Ang1*, *Vcam-1*, *Epo*, *Ccl2*, *Scf*, *Tpo*, *IL-6*, *Postn*, *Icam-1*, and *Sdf-1α* in splenic myofibroblasts in comparison to Lin⁻ splenic cells by qRT-PCR ($n = 3$–6). (**G**) Detection and comparison of transcript levels of known hematopoietic regulators *Ang1*, *Vcam-1*, *Epo*, *Ccl2*, *Scf*, *Tpo*, *IL-6*, *Postn*, *Icam-1*, and *Sdf-1α* in splenic myofibroblasts in comparison to Lin⁻ splenic cells by qRT-PCR; dCq values are compared for each gene ($n = 6$). (**H**) Effect of G-CSF treatment on the expression of *Sdf-1α* levels in Lin⁻ spleen cells and myofibroblasts. Quantitative RT-PCR was performed to quantify the transcript levels in the harvested cells and the graph shows relative change in the gene expression with and without G-CSF treatment ($n = 6, 8$). (**I**) Change in the transcript levels of *Ang1*, *Vcam-1*, *Epo*, *Ccl2*, *Scf*, *Tpo*, *IL-6*, *Postn*, *Icam-1*, and *Sdf-1α* in splenic myofibroblasts after G-CSF treatment. The gene expression level was quantified by performing qRT-PCR, and relative change in the abundance of transcripts following G-CSF treatment was plotted ($n = 6$). (**J**) Effect of G-CSF treatment on the transcript levels of *Ang1*, *Vcam-1*, *Epo*, *Ccl2*, *Scf*, *Tpo*, *IL-6*, *Postn*, *Icam-1*, and *Sdf-1α* in splenic myofibroblasts. The gene expression level was quantified by performing qRT-PCR, and the change in dCq values for each gene was examined following G-CSF treatment ($n = 6$). (**K**) Quantitative RT-PCR to examine the change in the transcript levels of *Ang1*, *Vcam-1*, *Epo*, *Ccl2*, *Scf*, *Tpo*, *IL-6*, *Postn*, *Icam-1*, and *Sdf-1α* in lineage-depleted splenic pulp cells after G-CSF treatment. Relative change in the abundance of transcripts is plotted ($n = 4$–6). (**L**) Change in the transcript levels of hematopoietic regulators in lineage-depleted splenic pulp cells after G-CSF treatment; dCq values before and after G-CSF treatment are compared for each gene ($n = 6$). Data is presented as bar graph (mean ± SEM) in panels (**F**, **G**, **I**–**L**) or box-whiskers plot (median with min to max) in panels (**B**, **C**, **H**). The *p*-value in figures (**B**, **C**, **F**–**L**) were calculated by the Student's *t*-test, $^*p < 0.05$, $^{**}p < 0.01$, $^{***}p < 0.001$, $^{****}p < 0.0001$, and ns $p > 0.05$, and ND indicates not detected.

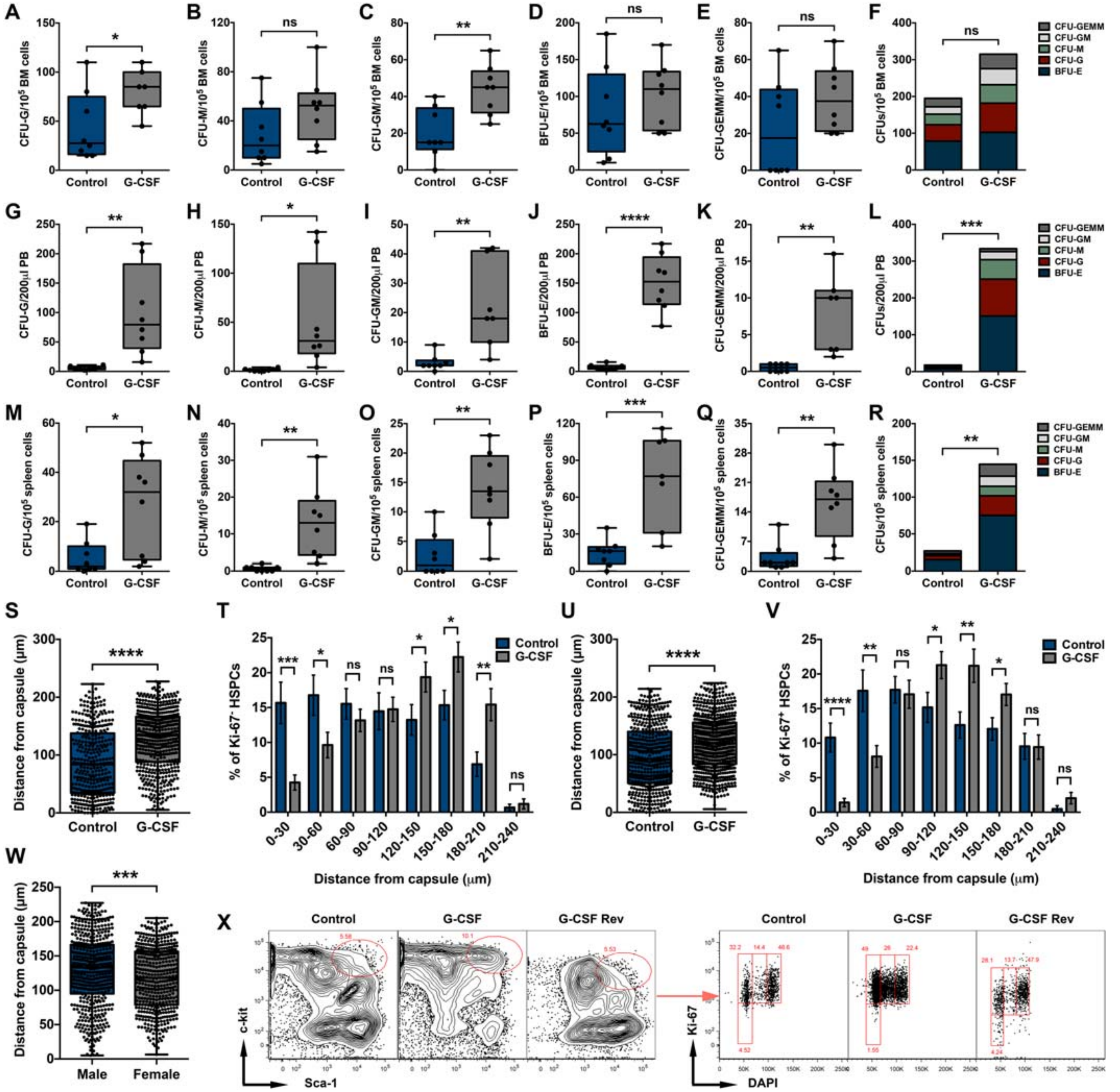
A
CFU-G/10^5 BM cells
Control
G-CSF
B
CFU-M/10^5 BM cells
C
CFU-GM/10^5 BM cells
D
BFU-E/10^5 BM cells
E
CFU-GEMM/10^5 BM cells
F
CFUs/10^5 BM cells
CFU-GEMM
CFU-GM
CFU-M
CFU-G
BFU-E
G
CFU-G/200µl PB
H
CFU-M/200µl PB
I
CFU-GM/200µl PB
J
BFU-E/200µl PB
K
CFU-GEMM/200µl PB
L
CFUs/200µl PB
M
CFU-G/10^5 spleen cells
N
CFU-M/10^5 spleen cells
O
CFU-GM/10^5 spleen cells
P
BFU-E/10^5 spleen cells
Q
CFU-GEMM/10^5 spleen cells
R
CFUs/10^5 spleen cells
S
Distance from capsule (µm)
T
% of Ki-67⁻ HSPCs
Distance from capsule (µm)
U
V
% of Ki-67⁺ HSPCs
W
Male
Female
X
Control
G-CSF
G-CSF Rev
c-kit
Sca-1
Ki-67
DAPI

© The Author(s)

Figure EV3. G-CSF treatment induces HSC mobilization and shift in HSCs away from capsular myofibroblasts.

(**A–F**) Methylcellulose based colony formation assays were performed to examine hematopoietic progenitor populations in the BM ($n = 7–8$). The frequency of (**A**) CFU-Gs, (**B**) CFU-Ms, (**C**) CFU-GMs, (**D**) BFU-Es, (**E**) CFU-GEMMs, and (**F**) total CFUs per 1×10^5 cells in the BM was compared with and without G-CSF treatment. (**G–L**) Comparison of the circulating hematopoietic progenitors in PB of mice treated with and without G-CSF ($n = 7–8$). The frequency of (**G**) CFU-Gs, (**H**) CFU-Ms, (**I**) CFU-GMs, (**J**) BFU-Es, (**K**) CFU-GEMMs, and (**L**) total CFUs in the MNCs harvested from 200 µl blood was plotted. (**M–R**) Colony formation assays were performed to examine the effect of G-CSF treatment on splenic hematopoietic progenitor populations ($n = 7–8$). The frequency of (**M**) CFU-Gs, (**N**) CFU-Ms, (**O**) CFU-GMs, (**P**) BFU-Es, (**Q**) CFU-GEMMs, and (**R**) total CFUs per 1×10^5 MNCs harvested from spleen tissues was compared. (**S**) Euclidean distances calculated for Ki-67$^-$ HSPCs (3$^-$c-kit$^+$ cells) relative to the nearest observable capsular myofibroblast of spleen tissues from control and G-CSF treated mice ($n = 6$, N; Control = 370, GCSF = 500 Ki-67$^-$ HSPCs). (**T**) Distribution of Ki-67$^-$ HSPCs at sequential distance intervals (30 µm each) relative to the capsular surface in spleen tissues with or without G-CSF treatment ($n = 6$, N; Control = 370, GCSF = 500 Ki-67$^-$ HSPCs). (**U**) Euclidean distances calculated for Ki-67$^+$ HSPCs relative to the capsular surfaces of spleen tissues treated with or without G-CSF ($n = 6$, N; Control = 466, G-CSF = 604 Ki-67$^+$ HSPCs). (**V**) Distribution frequency of Ki-67$^+$ HSPCs at sequential distance intervals relative to the capsular surface in spleen tissues with or without G-CSF treatment ($n = 6$, N; Control = 466, G-CSF = 604 Ki-67$^+$ HSPCs). (**W**) Euclidean distances calculated for each pHSC with reference to pseudo-surfaces of capsular myofibroblast in the spleens harvested from male and female mice after G-CSF treatment. Each dot represents a pHSC immunolocalized as a 3$^-$c-kit$^+$CD150$^+$ cell by confocal imaging ($n = 3$, N; male = 510, female = 447 pHSCs). (**X**) Flow cytometry analysis performed to analyze the cell cycle status of HSCs (Lin$^-$CD41$^-$CD48$^-$Sca-1$^+$c-kit$^+$ cell population) from the spleen tissues. Spleen tissues were harvested from control and G-CSF treated animals (same samples were used for data presented in Fig. 7E and Appendix Fig. S1H). G-CSF treatment was given for 5 days and the mice were sacrificed one day (G-CSF) or 30 days (G-CSF Rev) after the treatment. Data is presented as bar graph (mean ± SEM) in panels (**T**, **V**) or box-whiskers plot (median with min to max) in panels (**A–E**, **G–K**, **M–Q**, **S**, **U**, **W**) or stacked bars in panel (**F**, **L**, **R**). The p-value in figures (**A–W**) were calculated by the Student's t-test, *$p < 0.05$, **$p < 0.01$, ***$p < 0.001$, ****$p < 0.0001$, and ns $p > 0.05$.

© The Author(s)

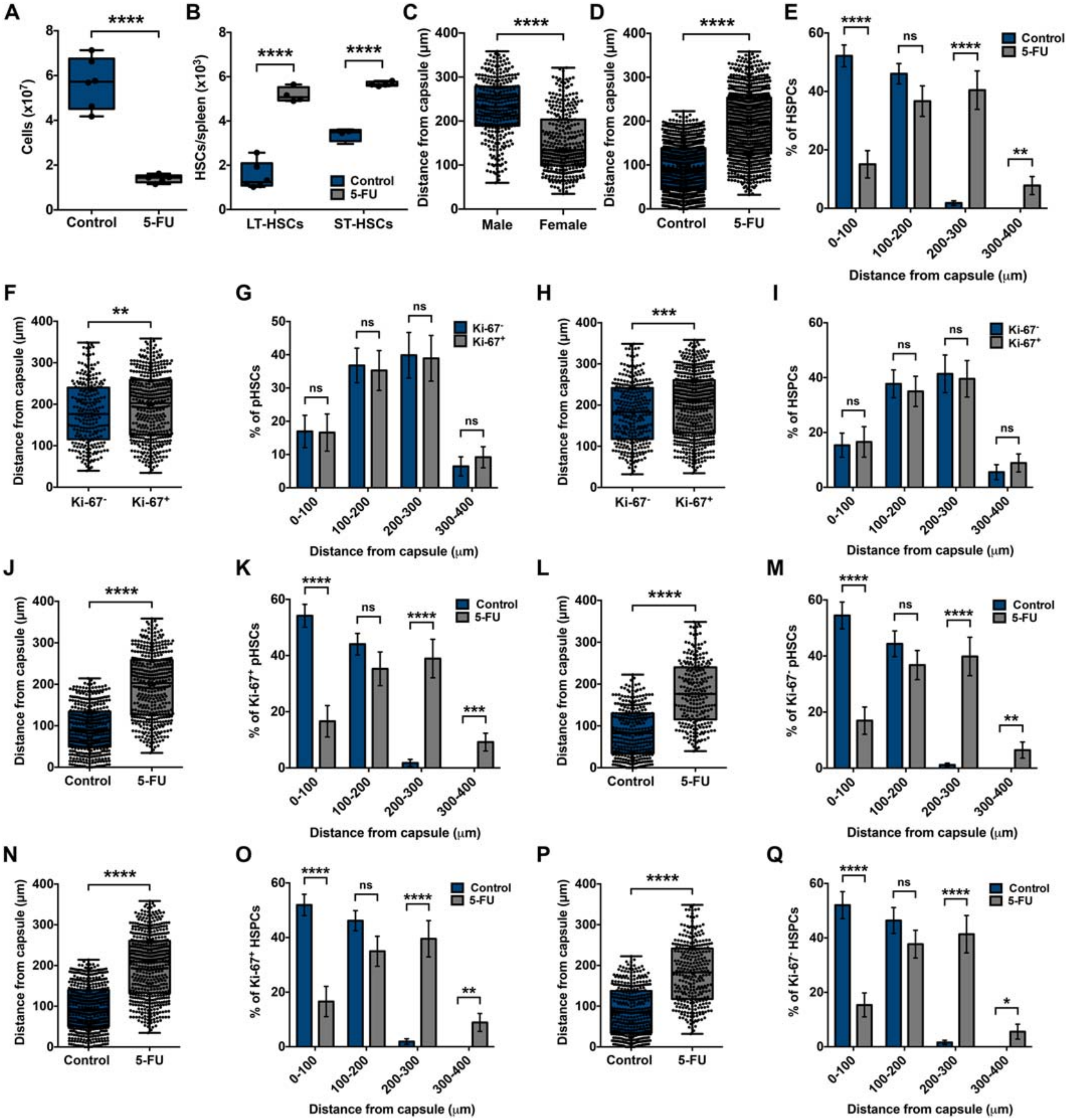
A
Cells (x10^7)
Control
5-FU
****
B
HSCs/spleen (x10^3)
LT-HSCs
ST-HSCs
Control
5-FU
C
Distance from capsule (µm)
Male
Female
D
Control
5-FU
E
% of HSPCs
0-100
100-200
200-300
300-400
Distance from capsule (µm)
F
Ki-67⁻
Ki-67⁺
G
% of pHSCs
H
I
J
K
% of Ki-67⁺ pHSCs
L
M
% of Ki-67⁻ pHSCs
N
O
% of Ki-67⁺ HSPCs
P
Q
% of Ki-67⁻ HSPCs

 © The Author(s)

Figure EV4. 5-FU mediated myeloablation reorganizes hematopoietic niche in spleen.

(**A**) Comparison of total spleen cellularity with and without 5-FU treatment. MNCs were harvested from the spleen tissues without enzymatic treatment, and viable cell counts were taken after RBC lysis using a Neubauer chamber ($n = 5$–6). (**B**) Comparison of total LT-HSC and ST-HSC in spleen tissues treated with and without 5-FU. Flow cytometry analysis was performed to identify and examine LT-HSC (Lin$^-$CD41$^-$CD48$^-$Sca-1$^+$c-kit$^+$CD150$^+$ cells) and ST-HSC (Lin$^-$CD41$^-$CD48$^-$Sca-1$^+$c-kit$^+$CD150$^-$ cells) populations in splenic MNCs harvested from control and 5-FU treated mice ($n = 4$–6). (**C**) Euclidean distances calculated for each pHSC with reference to pseudo-surfaces of capsular myofibroblasts in the spleen tissues harvested from male and female mice after 5-FU treatment. Each dot represents a pHSC immunolocalized as a 3$^-$c-kit$^+$CD150$^+$ cell by confocal imaging ($n = 2$, *N*; male = 327, female = 333; each dot represents a pHSCs). (**D**) Euclidean distances were calculated for each HSPC with reference to nearest capsular surface in the spleen with or without 5-FU treatment. Each dot represents a HSPC immunolocalized as a 3$^-$c-kit$^+$ cell by confocal imaging ($n = 4$–6, *N*; Control = 836, 5-FU = 796 HSPCs). (**E**) Distribution frequency of HSPCs at sequential distance intervals relative to capsular surface in the spleen tissues with or without 5-FU treatment ($n = 4$–6, *N*; Control = 836, 5-FU = 796 HSPCs). (**F**) Comparison of Euclidean distances for Ki-67$^-$ and Ki-67$^+$ pHSCs relative to the nearest capsular surface detected in spleen sections from 5-FU treated mice ($n = 4$, N; Ki-67$^-$ = 243, Ki-67$^+$ = 417 pHSCs). (**G**) Comparison between Ki-67$^-$ and Ki-67$^+$ pHSCs for their distribution frequency within sequential distance intervals relative to capsular surface in the spleen tissues after 5-FU treatment ($n = 4$, *N*; Ki-67$^-$ = 243, Ki-67$^+$ = 417 pHSCs). (**H**) Comparison of Euclidean distances for Ki-67$^-$ and Ki-67$^+$ HSPCs relative to the nearest α-SMA$^+$ myofibroblastic capsular surfaces in spleen sections from 5-FU treated mice ($n = 4$, *N*; Ki-67$^-$ = 300, Ki-67$^+$ = 496 HSPCs). (**I**) Comparison between Ki-67$^-$ and Ki-67$^+$ HSPCs for their distribution frequency within sequential distance intervals relative to capsular surface in the spleen tissues after 5-FU treatment ($n = 4$, *N*; Ki-67$^-$ = 300, Ki-67$^+$ = 496 HSPCs). (**J**) Euclidean distances were calculated for each Ki-67$^+$ pHSC with reference to nearest capsular surface in the spleen with or without 5-FU treatment. Each dot represents a Ki-67$^+$ pHSC immunolocalized by confocal imaging ($n = 4$–6, *N*; Control = 406, 5-FU = 417 Ki-67$^+$ pHSCs). (**K**) Distribution frequency of Ki-67$^+$ pHSCs at sequential distance intervals relative to capsular myofibroblastic surface in the spleen tissues with or without 5-FU treatment ($n = 4$–6, N; Control = 406, 5-FU = 417 Ki-67$^+$ pHSCs). (**L**) Comparison of Euclidean distances for Ki-67$^-$ pHSCs relative to the nearest capsular surfaces detected in spleen sections from control and 5-FU treated mice ($n = 4$–6, *N*; Control = 301, 5-FU = 243 Ki-67$^-$ pHSCs). (**M**) Comparison of Ki-67$^-$ pHSCs for their distribution frequency within sequential distance intervals relative to capsular surface in the spleen tissues with and without 5-FU treatment ($n = 4$–6, *N*; Control = 301, 5-FU = 243 Ki-67$^-$ pHSCs). (**N**) Euclidean distances calculated for Ki-67$^+$ HSPCs relative to the nearest observable capsular myofibroblastic surface of spleen tissues from control and 5-FU treated mice ($n = 4$–6, *N*; Control = 466, 5-FU = 496 Ki-67$^+$ HSPCs). (**O**) Distribution of Ki-67$^+$ HSPCs at sequential distance intervals relative to capsular surface in the spleen tissues with or without 5-FU treatment ($n = 4$–6, *N*; Control = 466, 5-FU = 496 Ki-67$^+$ HSPCs). (**P**) Euclidean distances calculated for Ki-67$^-$ HSPCs relative to the capsular surfaces of spleen tissues treated with or without 5-FU ($n = 4$–6, *N*; Control = 370, 5-FU = 300 Ki-67$^-$ HSPCs). (**Q**) Distribution frequency of Ki-67$^-$ HSPCs at different distance intervals relative to capsular surface in the spleen tissues with or without 5-FU treatment ($n = 4$–6, *N*; Control = 370, 5-FU = 300 Ki-67$^-$ HSPCs). Data is presented as bar graph (mean ± SEM) in panels (**E**, **G**, **I**, **K**, **M**, **O**, **Q**) or box-whiskers plot (median with min to max) in panels (**A–D**, **F**, **H**, **J**, **L**, **N**, **P**). The *p*-value in figures (**A–Q**) were calculated by the Student's *t*-test, $^*p < 0.05$, $^{**}p < 0.01$, $^{***}p < 0.001$, $^{****}p < 0.0001$, and ns $p > 0.05$.

© The Author(s)

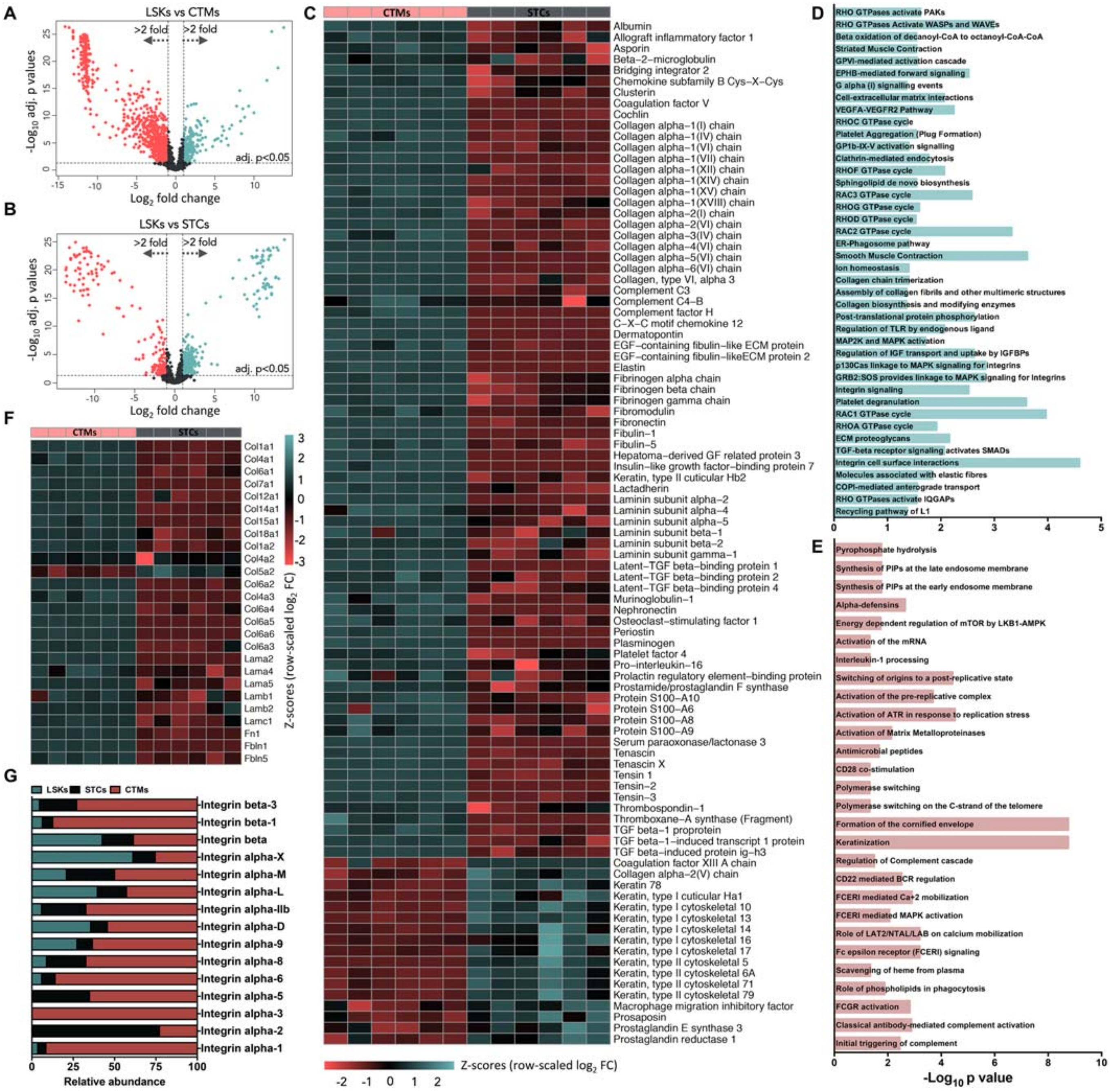

Figure EV5. Global proteome-based interactions show HSPC interactions preferential to myofibroblastic cells.

(**A**) Volcano plot showing proteins differentially enriched between LSK versus CTM populations. Log_2 fold change and $-log_{10}$ adjusted *p*-values are plotted on the x and y axes, respectively. Represented data are based on six independent biological replicates ($n = 6$), each with three technical replicates ($N = 18$). Cyan dots represent proteins with statistically significant (adjusted p-value < 0.05) higher abundance of >2 fold change. Red dots represent proteins having statistically significant (adjusted p-value < 0.05) lower abundance with >2 fold change. (**B**) Volcano plot showing differentially enriched proteins between LSK versus STC populations ($n = 6$, $N = 18$). (**C**) Heatmap analysis of differentially enriched secretory proteins in CTMs compared to STCs. The analysis was performed on the secretory proteins with differential enrichment with the adjusted *p*-values of <0.05 and fold change > 2.0. (**D**) Reactome pathway analysis of differentially enriched pathways based on proteins upregulated with fold change > 2.0 and adjusted p-value < 0.05. Significantly (p-value < 0.05) up-regulated pathways in CTMs versus STCs are illustrated. (**E**) Reactome pathway analysis was performed on proteins upregulated with fold change > 2.0 and adjusted p-value < 0.05. Differentially enriched pathways significantly (p-value < 0.05) up-regulated in STCs compared to CTMs are illustrated. (**F**) Heatmap analysis of differentially enriched secretory ECM proteins. Secretory ECM proteins with significantly (adjusted p-value < 0.05) altered enrichment (fold change > 2.0) between CTMs and STCs are illustrated. (**G**) Relative abundance of integrin sub-units enriched in LSKs, STCs, and CTMs. Represented data are based on six independent biological replicates ($n = 6$), each with three technical replicates ($N = 18$).

 © The Author(s)
